# Why large seeds with physical dormancy become nondormant earlier than small ones

**DOI:** 10.1371/journal.pone.0202038

**Published:** 2018-08-09

**Authors:** Ailton G. Rodrigues-Junior, Ana Caroline M. P. Mello, Carol C. Baskin, Jerry M. Baskin, Denise M. T. Oliveira, Queila S. Garcia

**Affiliations:** 1 Departamento de Botânica, ICB, Universidade Federal de Minas Gerais, Belo Horizonte, MG, Brazil; 2 Department of Biology, University of Kentucky, Lexington, KY, United States of America; 3 Department of Plant and Soil Sciences, University of Kentucky, Lexington, KY, United States of America; Brigham Young University, UNITED STATES

## Abstract

Under natural conditions, large seeds with physical dormancy (PY) may become water permeable earlier than small ones. However, the mechanism for this difference has not been elucidated. Thus, our aim was to evaluate the traits associated with PY in seeds of *Senna multijuga* (Fabaceae) and to propose a mechanism for earlier dormancy-break in large than in small seeds. Two seedlots were collected and each separated into large and small seeds. Seed dry mass, water content, thickness of palisade layer in the hilar and distal regions and the ratio between palisade layer thickness (P) in the lens fissure and seed mass (M) were evaluated. Further, the correlation between seed mass and seed dimensions was investigated. Large seeds had higher dry mass and water content than small seeds. The absolute thickness of the palisade layer in the different regions did not show any trend with seed size; however, large seeds had a lower P:M ratio than small seeds. Seed mass correlated positively with all seed dimensions, providing evidence for a substantially higher volume in large seeds. Since wet, but not dry, high temperatures break PY in sensitive seeds of *S*. *multijuga*, the data support our prediction that internal pressure potential in the seed and palisade layer thickness in the water gap (lens), which is related to seed mass (i.e. P:M ratio), act together to modulate the second step (dormancy break) of the two-stage sensitivity cycling model for PY break. In which case, large seeds are predetermined to become water-permeable earlier than small ones.

## Introduction

Water-impermeable seeds/fruits have physical dormancy (PY) and specialized structures that can open in response to environmental cues, thereby creating a ‘water-gap’ whereupon dormancy is broken [1–4]. The intensity of PY can vary between and within species, which may be related to (1) seed coat thickness, wherein a thick seed coat confers higher resistance to dormancy break [5–8]; (2) seed size, wherein large seeds become sensitive to environmental cues that break PY earlier than small ones [9–12]; and (3) seed water content during the acquisition of PY, which may result in differences in dormancy intensity [13–15]. However, the role of these features of the seeds in the dormancy breaking process is unclear.

A two-stage model for PY break was proposed by Taylor [16, 17] and Jayasuriya et al. [18, 19], wherein seeds cycle between insensitive and sensitive states (i.e. sensitivity cycling) [18, 19]. In the first step, seeds become sensitive to dormancy-breaking conditions, but they remain water-impermeable. If sensitive seeds are exposed to the appropriate dormancy-breaking conditions, they become water permeable, i.e. water-gap opens. On the other hand, if sensitive seeds are exposed to unfavorable dormancy-breaking conditions they revert to the nonsensitive condition [18, 19]. However, it is not known how seed coat thickness, seed size and seed water content are related to induction of sensitivity or to dormancy break in seeds with PY.

Baskin and Baskin [3, 14] did not subdivide the *class* PY into lower hierarchial categories in their seed dormancy classification scheme but suggested that it probably should be subdivided. Thus, more detailed information is needed to separate PY into *levels* and *types* [3, 19]. Based on seed water content during dispersal, Jaganathan [15] divided PY into two groups: shallow and absolute. The first group included seeds with a relatively high water content and a low intensity of PY, and the second group included seeds with a relatively low water content and a high level of dormancy. In addition to seed water content, seed size could affect PY. Rodrigues-Junior et al. [12] proposed a model for PY-break mediated by seed size, with large seeds being more sensitive to dormancy break than small seeds. These authors found that the seed size was a determining factor controlling the timing of dormancy-break (and thus germination) in seeds with PY, wherein small seeds require more time to complete the steps to break PY. Thus, in the first germination season more large seeds will germinate compared to small seeds [12]. Schutte et al. [8] suggested a possible trade-off between seed size and seed persistence in soil for species with PY, with persistence being directly related to seed coat thickness. Indeed, it is rather difficult to detect a relationship between seed coat thickness and level of dormancy, and Russi et al. [6] argued that measurements of the seed coat thickness in relation to seed size, as also evaluated in Schutte et al. [8], gives a more robust understanding of PY. They did not find a direct relationship between absolute seed coat thickness and dormancy.

As hypothesized by Russi et al. [6], the increase in seed volume accentuates the tension transmitted mechanically to the weak region (e.g. lens on legume seeds) of the seed coat in seeds with PY during expansion and contraction induced by environmental changes. Hence, a thinner seed coat is more susceptible to disruption than a thicker seed coat [6]. These authors suggested that the volume of seeds with greater mass varies more widely than that of seeds with less mass when exposed to temperature fluctuations during the year and thus dormancy break would occur more quickly in large than in small seeds. Furthermore, seeds of some legume species at different positions within the fruit may exhibit a sequence of PY-break that is associated with the seed size [9, 10]. Based on differences in seed mass before and after approximately 2 years on bare soil, Smith et al. [11] suggested that small seeds persist in soil longer than large ones. However, no study has investigated in detail the effects of seed size and mass in relation to thickness of the palisade layer on the susceptibility to PY break.

Thus, we hypothesized that seed size and mass are correlated with seed water content and thickness of the water-impermeable palisade layer in the seed coat and that these features are related to PY break. Since seed water content can be related to the intensity of PY [15], an increase in seed volume accentuates the tension on the seed coat and a thinner seed coat is more susceptible to disruption [6], we predicted that large seeds have higher water content and a thinner palisade layer than small seeds, thus explaining the differences between sizes on breaking PY. To test our hypothesis, we used seeds of *Senna multijuga*, a species with physically dormant seeds in which seed size mediates the time of response to the environmental cues during the PY-breaking process [12]. In this species, seeds made sensitive after exposure to temperatures ≤ 20°C become water-permeable at a high temperature (35°C) on a moist substrate. However, large seeds need a shorter period of time to complete these two steps in PY-break, and thus they germinate earlier than small ones during the growing season. Thus, our aims were to (1) identify the features of large and small seeds of *S*. *multijuga* that may be involved in the breaking dormancy process, and (2) discuss these features in relation to the second-step of PY-break in seeds in response to summer habitat conditions.

## Materials and methods

### Seed collection and processing

Seeds were manually collected from dry fruits at two locations on the campus of the Universidade Federal de Lavras, Brazil [seed collection 1 (S1) (21° 13′ 39,34″ S, 44° 58′ 11,85″ W; seed collection 2 (S2) (21° 13′ 30,53″ S, 44° 58′ 27,12″ W)] in September 2014 from 12 individuals for each collection. The location where S1 seeds were collected is about 1 km from where the S2 seeds were collected. Thus, the climate in these two locations is similar. Average temperature (max/min) is around 30/20°C during the spring/summer and 25/15°C during autumn/winter (Instituto Nacional de Meteorologia–INMET, Brazil). However, a clear distinction between S1 and S2 is the age of plants from which seeds were collected. S1 plants were older than S2, which has been shown to have an effect on germination [14]. Nonfilled seeds were discarded after flotation in water. Seeds were then blotted dry and placed in plastic trays in ambient room conditions [25±5°C, 40–60% relative humidity (RH)] for 24 h. Then, the seeds were stored in sealed semipermeable plastic bags in the same conditions until the beginning of the experiments 1 year later. This storage period does not break PY in *S*. *multijuga* seeds [12]. These two seed lots also were used in the study by Rodrigues-Junior et al. [12], who found that seeds in the S1 collection were larger than those in the S2 collection. Further, large S1 and S2 seeds became sensitive faster than small S1 and S2 seeds, and thus large seeds germinated earlier in the field than small seeds [12].

### Seed dry mass and water content

**Fig 1:**
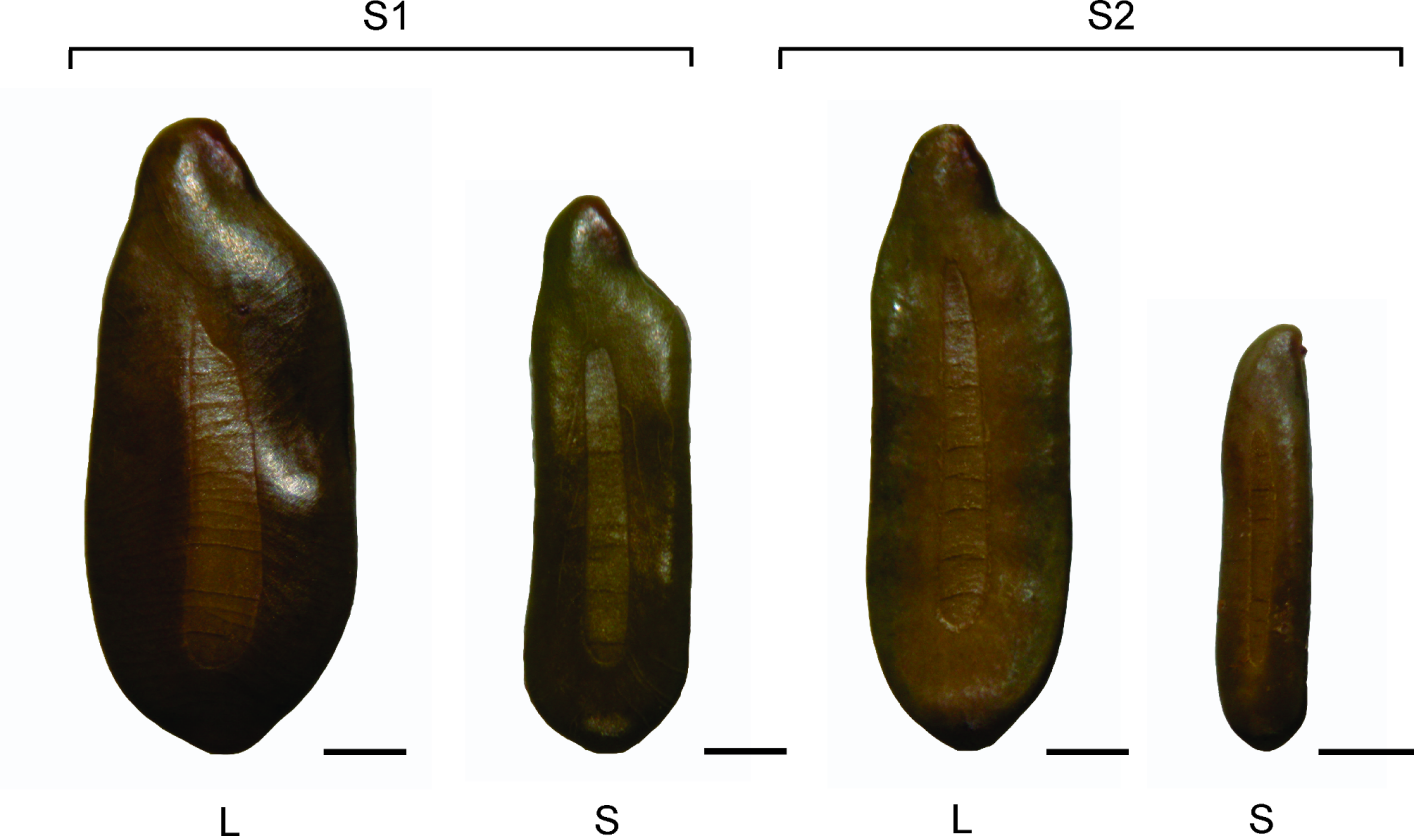
Large (L) and small (S) *Senna multijuga* seeds from collections S1 and S2. Bars = 1 mm.

Firstly, S1 and S2 seeds were separated into two groups: (1) large seeds and (2) small seeds (Fig 1). To do this, seeds of each collection were first visually separated into large and small (since the difference regarding seed sizes is evident), and then seeds from each group were individually weighed. The mass of large S1 seeds was > 0.02 and that of small S1 seeds < 0.016 g, and the mass of large S2 seeds was > 0.01 g and that of small S2 seeds < 0.008 g. These two groups were separated since the difference between these two sizes in relation to breaking PY and consequent germination is quite clear (Rodrigues-Junior et al., [12]). To determine seed water content and dry mass, 25 seeds from each of the four groups (two seed sizes x two seed collections) were scarified with sandpaper to allow water loss during drying, weighed individually using a Shimadzu AUX220 analytical balance (0.00001 g), oven-dried at 103°C for 17 hours and then weighed again. The data for seed water content were expressed as percentage of water on a fresh weight basis [20].

### Relationship between thickness of palisade layer and seed size and mass

**Fig 2:**
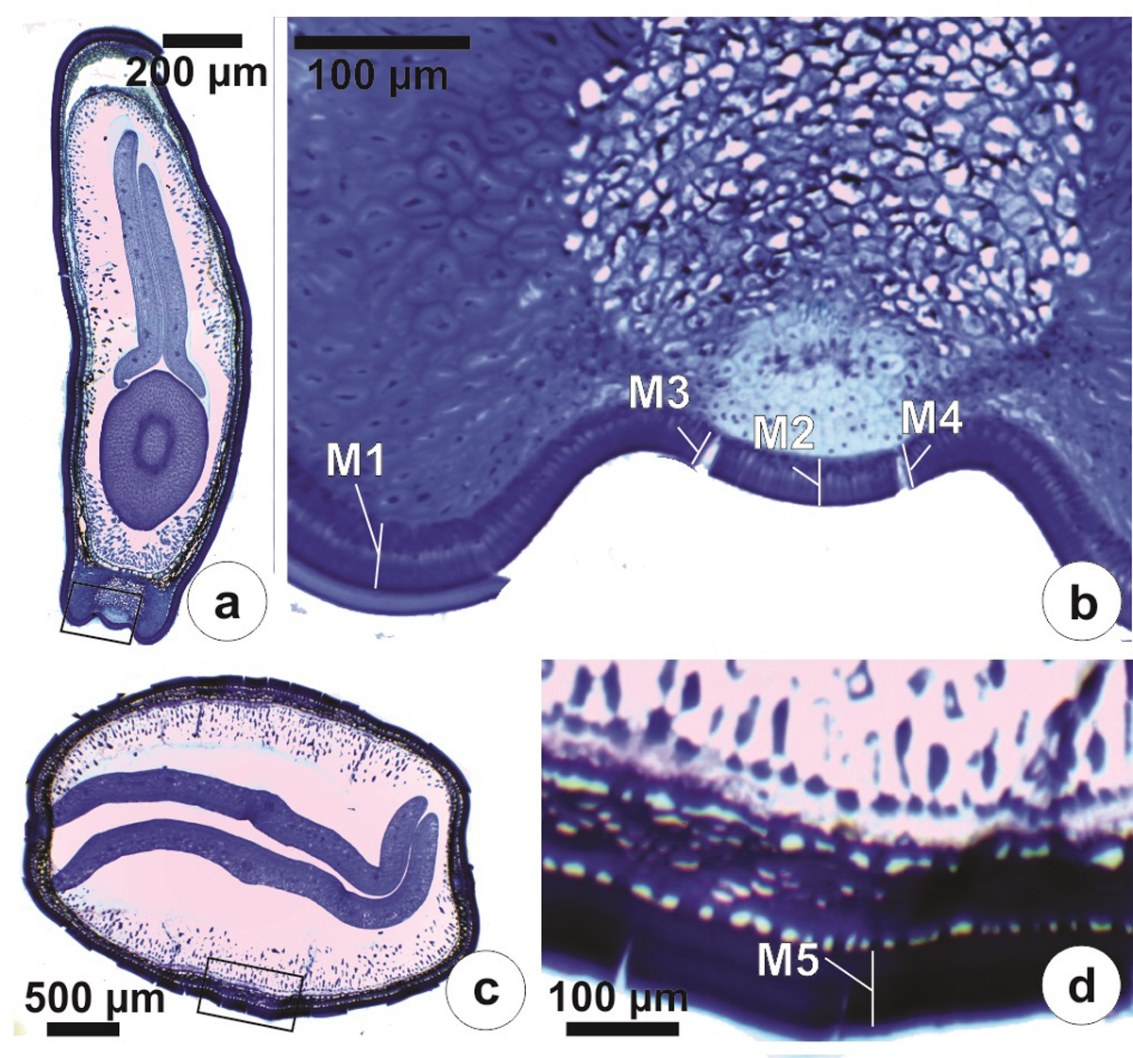
Sections of *Senna multijuga* seeds showing locations where palisade layer was measured. (A) Cross-section in hilar region. (B) Detail of region indicated by rectangle in A. (C) Cross-section in distal region (lateral position of the median third). (D) Detail of the region indicated by the rectangle in C. M1–M5 = locations where measurements were made on each seed.

Thickness of the palisade layer in the hilar and in the extra-hilar (in the middle third of the lateral part) regions was measured for large and small S1 and S2 seeds. Seeds were made water-permeable by immersing them in hot water (80°C for 15 min) [21]. Then, seeds were fixed with FAA for 48 h, dehydrated in a graded ethanol series and infiltrated with and embedded in 2-hydroxyethyl-methacrylate. Seed material was sectioned (8 μm) transversally using a Zeiss Hyrax M40 microtome, stained with 0.05% toluidine blue, pH 4.7 (modified from O’Brien et al. [22]) and mounted in synthetic resin. Sections were observed using a Leica DM500 optical microscope and photographs taken with a Leica ICC50 HD digital camera. Five measurements were made on each seed, using 10 replicates for each of the two sizes of S1 and S2 seeds. Thickness of the palisade layer was measured on the lateral part of the hilar region (M1), in the middle of the lens (M2) and on the two sides (lateral position) of the lens where a split had occurred (M3, M4). Thickness of the palisade layer was also measured in the distal (extra-hilar) region (M5) (Fig 2). The average for M3 and M4 was used to determine the mean thickness of the palisade layer in the lens split. The ratio between thickness of the palisade layer (P) in the lens fissure and seed fresh mass (M) was calculated (P:M ratio) based on Russi et al. [6] and Schutte et al. [8].

### Relationship between seed mass and seed dimensions

To assess the relationship between seed mass and seed length, width and thickness, S1 and S2 seeds were randomly sampled, and these three dimensions and seed mass were measured for individual seeds. For this assay, we used seeds of all sizes, i.e. large, small and those on the gradient between large and small, in the two collections. The three dimensions were measured using Mitutoyo 500-144B digital calipers, and fresh mass of each seed was determined using the Shimadzu AUW220D analytical balance. One-hundred seeds (50 S1 and 50 S2) were used. Then, the relationship between each seed dimension and seed mass was analysed.

### Statistical analyses

The data for seed dry mass and water content, palisade layer measurements and P:M ratio were firstly tested in separate models for normality (Shapiro-Wilk test) and homoscedasticity (Barlett test) (*P*≥0.05) to verify that they fit the assumptions of ANOVA. Since the data were nonparametric, they were analysed with a generalized linear model (GLM) with binomial distribution, and the means were compared by the post-hoc LSD test at 5% probability using R software for Windows [23]. The statistical models included the effects of seed collection and seed size as well as their interactions. Regression analyses were applied to evaluate the relationship between seed mass and seed dimensions. All graphs were designed using SigmaPlot^®^ software (Systat Software Inc., San Jose, California, USA).

## Results

### Seed dry mass and water content

**Fig 3:**
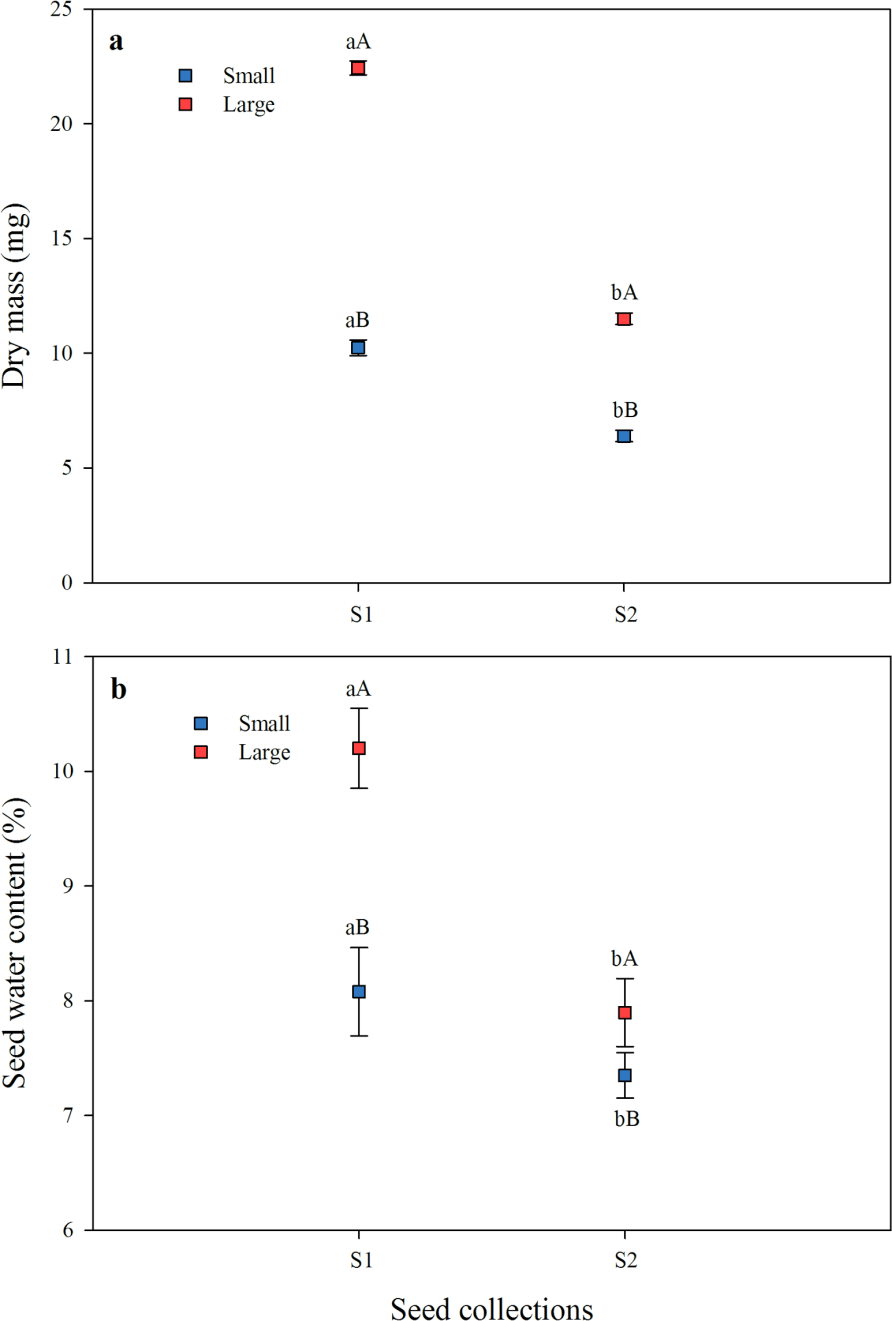
**Mean (± s.e.) dry mass (A) and water content (B) of small and large S1 (seed collection 1) and S2 (seed collection 2) seeds.** Different lowercase letters indicate significant differences between seed collections within a seed size and different uppercase letters significant differences between seed sizes within a collection, according to Fisher’s test (*P*≤0.05).

Large seeds had higher dry mass than small seeds in both collections. Small S1 and large S1 seeds had higher dry mass than small S2 and large S2 seeds, respectively (*P*<0.001) (Fig 3A). Large seeds had higher water content than small seeds in both collections. Small S1 and large S1 seeds had higher water content than small S2 and large S2 seeds, respectively (*P*<0.001) (Fig 3B).

### Relationship between thickness of palisade layer and seed size and mass

**Fig 4:**
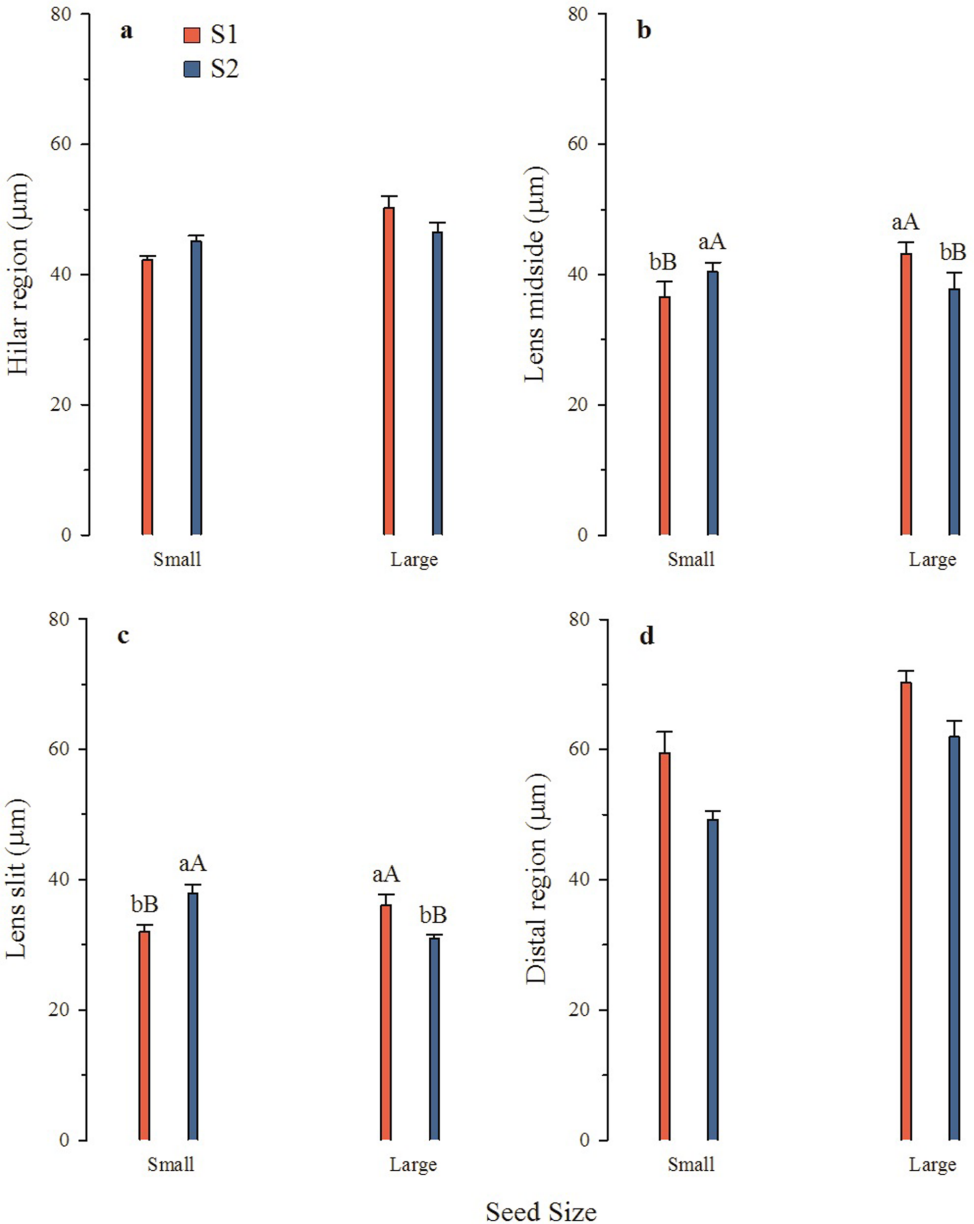
Thickness of palisade layer (mean ± s.e.) in different parts of S1 (seed collection 1) and S2 (seed collection 2) seeds. Different lowercase letters indicate significant differences between seed sizes within a collection and different uppercase letters significant differences between seed collections within a seed size, according to Fisher’s test (*P*≤0.05). There was no interaction between seed size and seed collection in A and D.

There was no interaction between seed collection and seed size in the measurements at the hilar region, and only seed size affected thickness of the palisade layer in the hilar region (*P* = 0.03) (Fig 4A). There was an interaction between seed size and seed collection for thickness of the palisade layer in the lens (in middle region) (*P* = 0.02), but no trend was found for these measurements. Large S1 seeds had a thicker palisade layer than small seeds, and small S2 seeds had a thicker palisade layer than large seeds (Fig 4B). For thickness of the palisade layer in the slits in the lens, there was an interaction between seed size and seed collection (*P*<0.001). In the slit region, large S1 seeds had a thicker palisade layer, whereas small S2 seeds were thicker in this region. Small S2 had a thicker palisade layer in the slit region than small S1 seeds. S1 large seeds had a thicker palisade layer in the lens slit than S2 large seeds (Fig 4C). Seed size (*P*<0.001), and seed collection (*P*<0.001) affected thickness of the palisade layer in the distal region. Large seeds had a thicker palisade layer in the distal region than small ones in both seed collections, and S1 seeds had a thicker palisade layer in the extra-hilar region than S2 seeds (Fig 4D).

**Fig 5:**
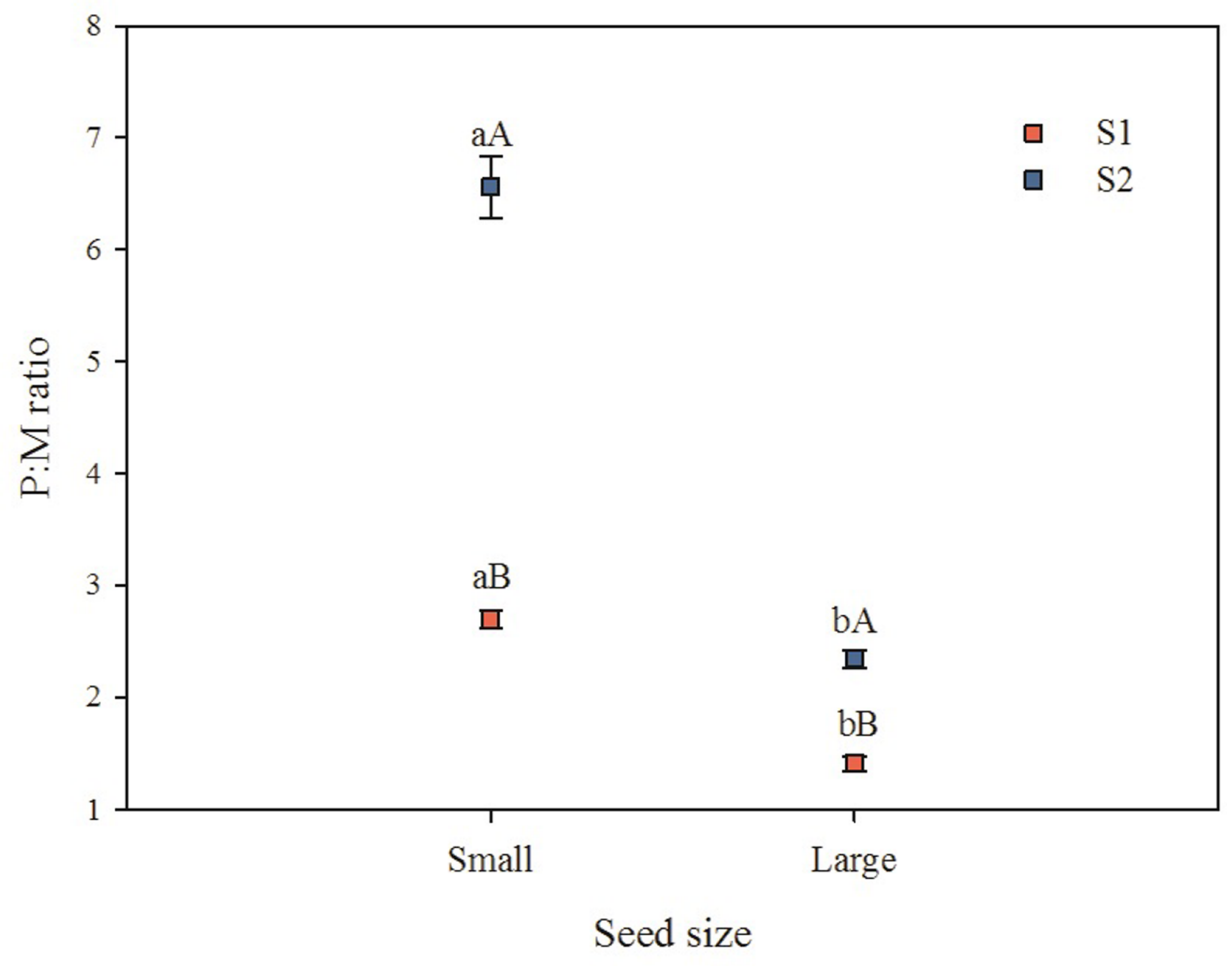
Relationship between P:M ratio and seed size for S1 (seed collection 1) and S2 (seed collection 2) seeds (mean ± s.e.). Different lowercase letters indicate significant differences between seed sizes within a collection and different uppercase letters significant differences between seed collections within a seed size, according to Fisher’s test (*P*≤0.05).

There was an interaction between seed size and seed collection (*P*<0.001) for the P:M ratio, and both S1 and S2 small seeds had a higher ratio than large seeds. S1 large and small seeds had a lower P:M ratio than S2 large and small seeds, respectively (Fig 5).

### Relationship between seed mass and seed dimensions

**Fig 6:**
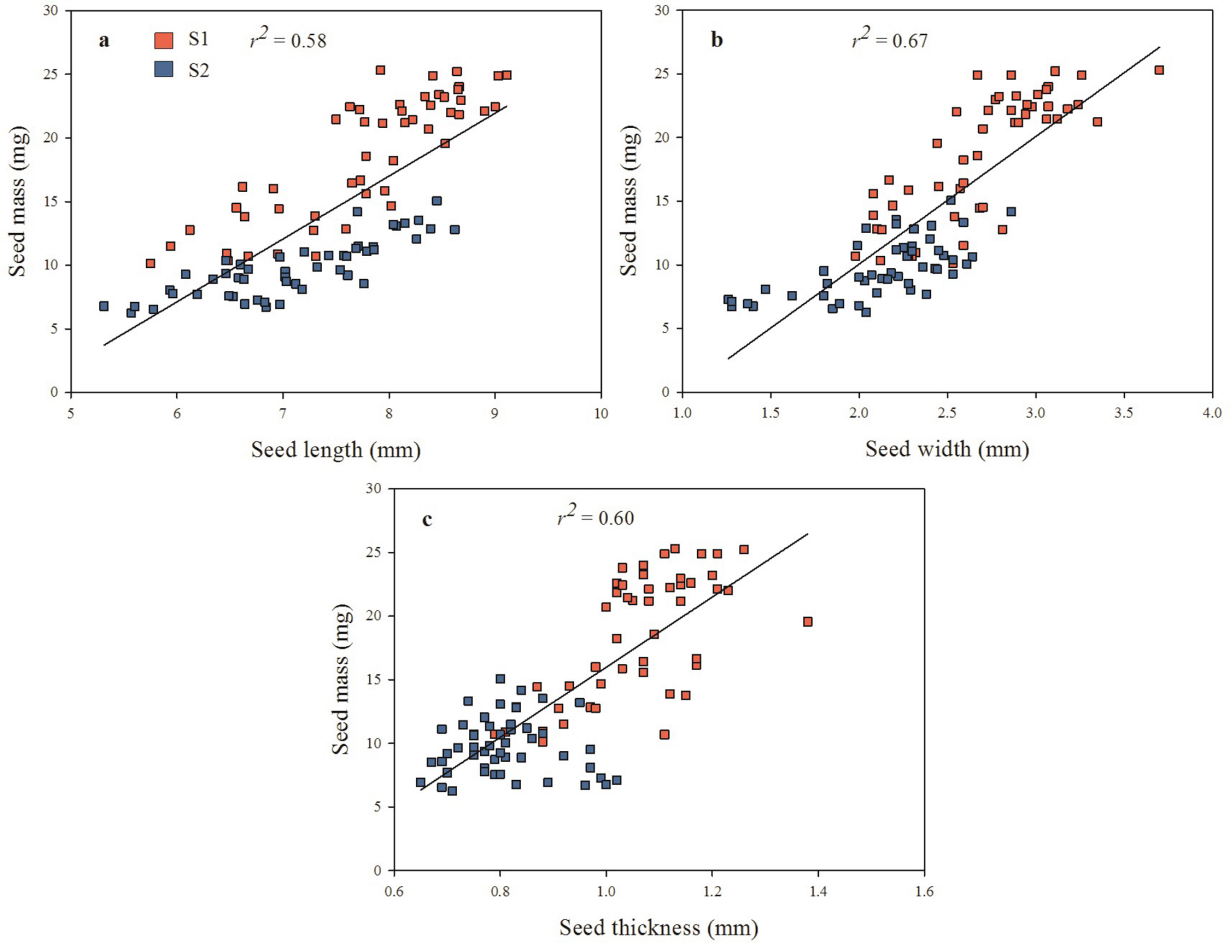
**Relationship between seed mass and (A) seed length, (B) seed width and (C) seed thickness.** (*n* = 100, all *P* < 0.0001).

Seed dimensions increased with seed mass (Fig 6A–6C). All of these seed parameters were strongly and positively related to seed mass (*P*<0.0001). That is, with an increase in seed length, width or thickness there was an increase in seed mass. Also, S1 seeds had more mass than those of S2 (Fig 6A–6C).

## Discussion

Large seeds of *S*. *multijuga* had higher dry mass and higher water content than small seeds, which is what we predicted. However, contrary to our predictions, there was no trend in the relationship between absolute thickness of palisade layer and seed size. On the other hand, large seeds had a low P:M ratio, while small seeds had a high P:M ratio. Thus, the relative thickness of the palisade layer (when seed mass was taken into account) indicated differences between large and small seeds. Also, S2 (collection with smallest seeds) had a higher P:M ratio than S1 seeds. There is a direct relationship between seed mass and seed dimensions in *S*. *multijuga*, and thus an increase in seed volume occurs with an increase in seed mass. All of these results support the seed size-mediated model for PY-break proposed by Rodrigues-Junior et al. [12]. In this model, small seeds need more time to complete the two steps to break PY than large seeds, spreading germination over time.

To explain differences in relation to PY break among *Acacia* species, Venier et al. [24] found that seeds with a thin seed coat were responsive to dormancy-break during gut passage through cattle, while those with thick seed coat remained dormant. These results could indicate that absolute seed coat thickness is associated with dormancy relief. However, for other species this association is not clear [6, 8]. Since different seeds have distinct physical properties, it is difficult to make comparison among species. On the other hand, when the comparison is made within a species there is a clear pattern for the breaking of PY, with large seeds becoming nondormant earlier than small ones [9, 10]. Thus, to understand why this pattern occurs in physically dormant seeds, we need to gather information about breaking PY in seeds.

For some species, an increase in internal vapour pressure affects the breaking of PY [25]. This occurs for seeds of *Ipomoea lacunosa* (Convolvulaceae) [25]. In this species, the increase in the internal vapour pressure is caused by absorption of water vapour through the fissure formed in the hilum in sensitive seeds, which closes after water vapour absorption. Thus, hilar closure prevents loss of internal water, and thus vapour pressure increases with an increase in temperature. *Senna multijuga* seeds require the same conditions (wet substrate, 35°C) [12] as those of *I*. *lacunosa* to break dormancy [25], and the fissures formed in the hilum could act in the same way, thus contributing to an increase in seed internal pressure when subjected to high temperatures. High temperatures may elevate the internal energy and exert force on the seed coat, which disrupts in the weakest region of the seed coat, namely the lens. *Senna multijuga* seeds remain water-impermeable at temperatures lower than 35°C [12]. Therefore, if the model proposed by Jayasuriya et al. [25] fits *S*. *multijuga* seeds, why is dormancy in large seeds broken earlier/faster than that in small ones? The higher water content of large than of small seeds and thus more water per volume may increase internal vapour pressure more in large than in small seeds. Therefore, with exposure to high temperatures enough force is generated in large seeds to move the palisade layer outward in the weak region in the lens. In fact, the role of internal pressure on the mechanism to break PY was first mentioned by Hanna [26], but this author suggested that a possible increase in pressure caused by heat treatment could be due to an increase in the number of vascular bundle below the lens for *Acacia kempeana* seeds. In fact, Fig 3B shows that the water content of large S2 seeds was not significantly different from that of small S1 seeds. However, this is explained by the differences in seed size in each collection. That is, small S1 and large S2 seeds are quite similar, as described above.

Hanna [26] and Serrato-Valenti et al. [27] found an evident weak region in the lens in *Acacia kempeana* and *Leucaena leucocephala* seeds (Fabaceae, Mimosoideae). This weak region was related to a decrease in height of cells in the palisade layer, but it was not determined if changes in cell height were related to the dormancy level in these species. We also found a decrease in height (thickness) of cells in the palisade layer on the two sides of the lens where a split occurs. However, in *S*. *multijuga* thickness of the palisade layer is related to the propensity for breaking dormancy only when seed mass is taken into account. Thus, in addition to the internal pressure in the seeds, thickness of the palisade layer may affect the PY-breaking mechanism by providing physical resistance to the force exerted by internal pressure, and seed size can modulate this mechanism.

Seed water content at the onset of dormancy in *S*. *multijuga* is related to seed size, and it determines whether dormancy is broken earlier or later in the growing season. Large seeds may lose less moisture than small ones because they accumulate a larger amount of dry mass, and the distance from the distal region of the seed to the hilum (region where the moisture moves out of the seed) is greater than that in small seeds. Consequently, large seeds tend to have higher water content at equilibrium with the surrounding environment than small seeds. This conclusion agrees with Hyde’s [28] statement that “The duration of the impermeable condition increased with the degree of desiccation brought about by loss of water through the hilum”. Hyde [28] also demonstrated that exposing dormant seeds to gradually increasing humidity can manipulate the mechanism of water control by the hilum and increase seed water content. In the conditions tested, an increase in water content was associated with an increase in germination [28].

**Fig 7:**
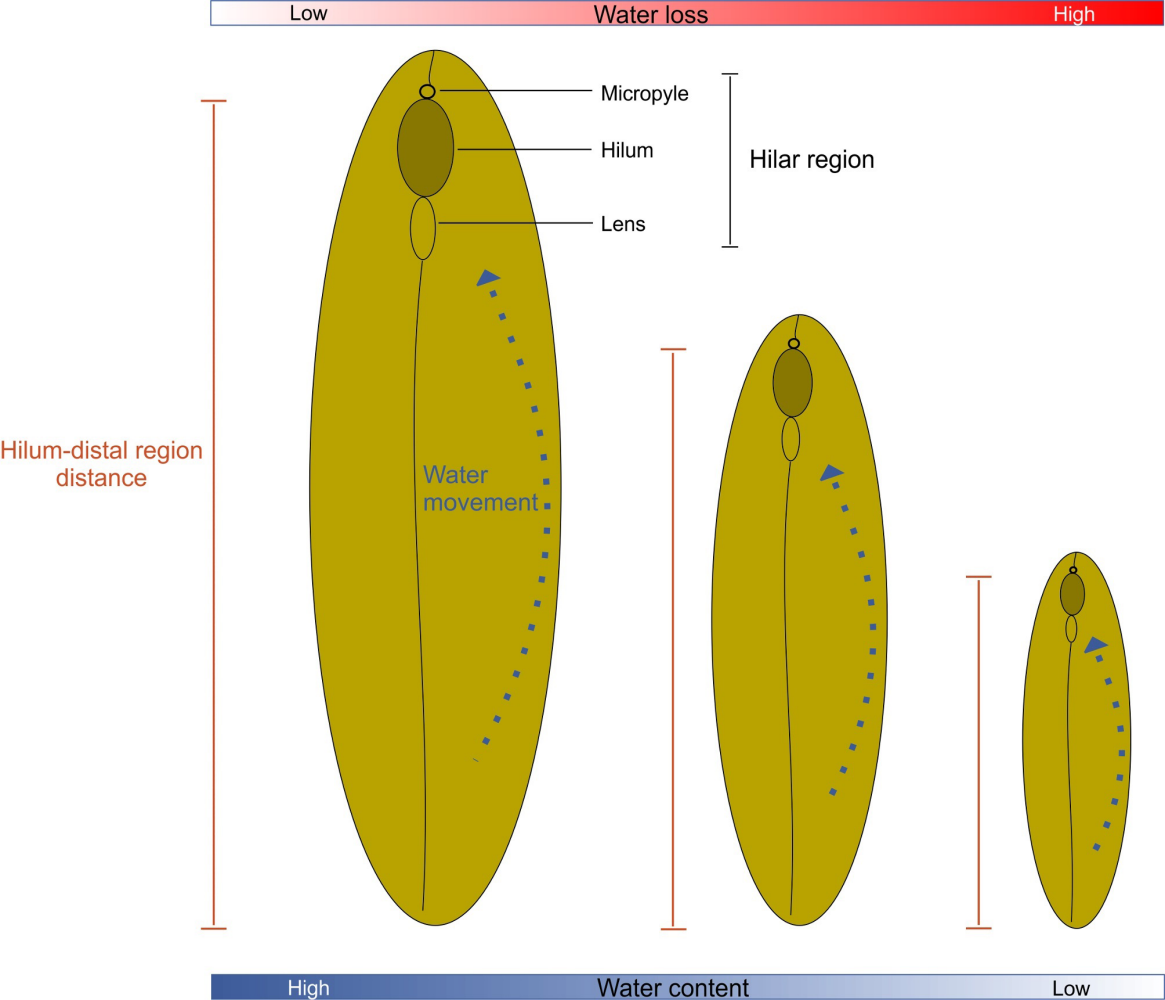
Conceptual model of differences in water loss during onset of PY in relation to seed size. Blue dotted arrows indicate path of water inside the seed towards the hilar region. Red scale represents the variation in water loss among the sizes of seeds. Blue scale represents the variation in water content among the sizes of seeds.

The consequent increase in seed volume in large seeds could affect the rate of water loss during the acquisition of PY. That is, in large seeds there is a reduction in the amount of water lost since the cell-to-cell water transport towards the hilum requires more time in large than it does in small seeds. Furthermore, Hyde [28] demonstrated that the water content of dormant seeds (with PY) equilibrates with the lowest relative humidity in the environment surrounding the seed. However, we found a difference in water content between large and small *S*. *multijuga* seeds collected from the field. A greater resistance to further dehydration in large than in small seeds allows the maintenance of a higher water content in large than small seeds. Thus, we propose a conceptual model for the differences in water loss in relation to seed size (Fig 7).

A relationship has been found between seed size and PY, wherein small seeds tend to be more dormant than large ones [6, 8, 10, 12]. However, since PY is coat-imposed the differences in dormancy are caused by variation in the ability to open the water gap. Resistance of the water gap to disrupt (seed coat thickness) plus internal force (pressure) act during the process of breaking physical dormancy. With an increase in the thickness of the seed coat, the force required to open the water gap increases. This relation is true in the case of physically dormant seeds that need moisture to break dormancy. The role of internal pressure in breaking PY was hypothesized by Jayasuriya et al. [25, 29], who observed a distinct response to wet and dry conditions for seeds of congeneric species of Convolvulaceae to become permeable. Similarities are shared by *S*. *multijuga* and *Ipomoea lacunosa* seeds during the second step of dormancy break, as evidenced by Rodrigues-Junior et al. [12], and both species require summer habitat conditions to become water-permeable. The requirement for wet-high temperatures to break PY in *S*. *multijuga* seeds and the relationship between seed traits of this species support the role of internal pressure in the PY-breaking mechanism in seeds proposed by Jayasuriya et al. [25, 29].

Large *S*. *multijuga* seeds have a higher water content and a lower P:M ratio than small seeds. That is, the impermeable barrier can be broken in the weak region of the lens earlier in large seeds than in small ones, which explains why large seeds germinated earlier than small ones in the study by Rodrigues-Junior et al. [12]. Therefore, the PY-breaking mechanism is much more complex than a simple retraction and expansion of the seed coat. The relationship between internal pressure potential and the relative palisade layer thickness in the water gap (lens in this case) is related to seed size, and jointly they modulate the second step of the two-stage model for PY break proposed by Taylor [16, 17] and Jayasuriya et al. [18, 19].
